# 1,6- and 1,7-Regioisomers of Asymmetric and Symmetric Perylene Bisimides: Synthesis, Characterization and Optical Properties

**DOI:** 10.3390/molecules19010327

**Published:** 2013-12-27

**Authors:** Hsing-Yang Tsai, Che-Wei Chang, Kew-Yu Chen

**Affiliations:** Department of Chemical Engineering, Feng Chia University, Taichung 40724, Taiwan; E-Mails: b77315@hotmail.com (H.-Y.T.); a1981207@hotmail.com (C.-W.C.)

**Keywords:** 1-amino-6-nitroperylene bisimide, 1-amino-7-nitroperylene bisimide, 1,6-diaminoperylene bisimide, 1,7-diaminoperylene bisimide, asymmetric perylene bisimides, symmetric perylene bisimides

## Abstract

The 1,6- and 1,7-regioisomers of dinitro- (1,6-**A** and 1,7-**A**) and diamino-substituted perylene bisimides (1,6-**B** and 1,7-**B**), and 1-amino-6-nitro- and 1-amino-7-nitroperylene bisimides (1,6-**C** and 1,7-**C**) were synthesized. The 1,6-**A** and 1,7-**A** regioisomers were successfully separated by high performance liquid chromatography and characterized by 500 MHz ^1^H-NMR spectroscopy, and subsequently, their reduction which afforded the corresponding diaminoperylene bisimides 1,6-**B** and 1,7-**B**, respectively. On the other hand, the monoreduction of 1,6-**A** and 1,7-**A**, giving the asymmetric 1-amino-6-nitro (1,6-**C**) and 1-amino-7-nitroperylene bisimides (1,7-**C**), respectively, can be performed by shortening the reaction time from 6 h to 1 h. This is the first time the asymmetric 1,6-disubstituted perylene bisimide 1,6-**C** is obtained in pure form. The photophysical properties of 1,6-**A** and 1,7-**A** were found to be almost the same. However, the regioisomers 1,6-**C** and 1,7-**C**, as well as 1,6-**B** and 1,7-**B**, exhibit significant differences in their optical characteristics. Time-dependent density functional theory calculations performed on these dyes are reported in order to rationalize their electronic structure and absorption spectra.

## 1. Introduction

Perylene bisimides (PBIs) and their related derivatives have received considerable attention due to their potential applications in molecular electronic and optical devices, such as light-emitting diodes [1,2,3,4], organic field-effect transistors (OFETs) [5,6,7,8,9,10], light-harvesting arrays [11,12], photovoltaic cells [13,14,15,16,17,18,19,20,21,22], LCD color filters [23,24], photochromic materials [25,26], and molecular wires [27,28]. PBIs have also been utilized as building blocks to construct supramolecular or artificial photosynthetic systems [29,30,31]. These compounds are advantageous due to their high molar absorptivities, excellent thermal and optical stabilities, ease of synthetic modification and reversible redox properties [32,33,34,35,36,37,38,39,40,41,42]. Additionally, the electronic characteristics of PBIs can be fine-tuned by the substitution of the conjugated aromatic core. Based on this concept, more and more perylene bisimide derivatives with either electron-donating or electron-withdrawing groups have been reported in the literature, including: (a) piperidinyl-substituted PBIs [43,44,45]; (b) pyrrolidinyl-substituted PBIs [46,47,48]; (c) alkylamino-substituted PBIs [49,50,51]; (d) amino-substituted PBIs [52,53]; (e) alkoxy-substituted PBIs [54,55,56,57,58]; (f) hydroxy-substituted PBIs [59,60]; (g) aryl-substituted PBIs [61,62]; (h) ferrocenyl-substituted PBIs [63,64]; (i) alkyl-substituted PBIs [65]; (j) perfluoroalkyl-substituted PBIs [66,67]; (k) boryl-substituted PBIs [68]; (l) cyano-substituted PBIs [69,70]; (m) nitro-substituted PBIs [71,72,73], *etc*.

**Scheme 1:**
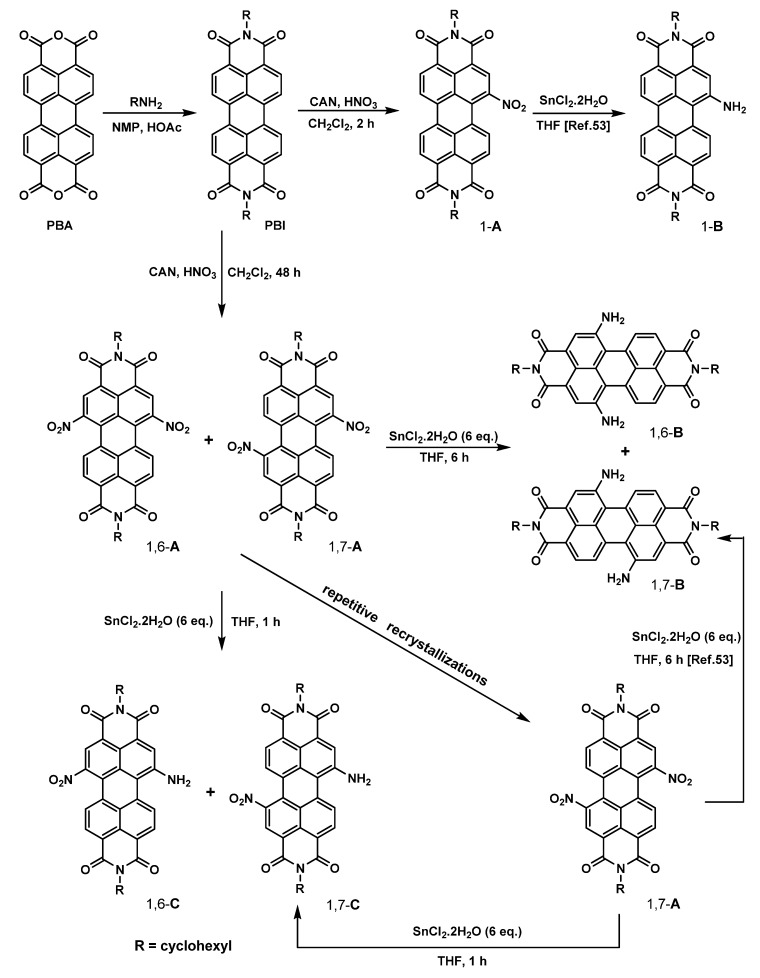
The synthetic routes to **A**–**C** [53].

To date, a general strategy for introducing substituents onto the PBIs core is bromination of perylene dianhydride. Subsequently, nucleophilic substitutions and metal-catalyzed cross-coupling reactions can then be performed and yield a regioisomeric mixture of 1,7- (major) and 1,6-disubstituted (minor) PBIs. However, only a few reports of isolation and characterization of both 1,6- and 1,7-disubstituted PBIs have been reported, and still very little is known about the spectroscopic properties of 1,6-disubstituted PBIs [43,46,63]. To expand the scope of PBI-based chromophores available for designing systems for colorful dyes and charge transport, we have recently [53] synthesized a series of symmetric 1,7-dinitro- and 1,7-diaminoperylene bisimides (1,7-**A** and 1,7-**B**, Scheme 1). Herein, we present our research for the synthesis, separation, characterization, optical properties, and complementary time-dependent density functional theory (TD-DFT) calculations of 1,6- and 1,7-regioisomers of asymmetric and symmetric PBIs.

## 2. Results and Discussion

### 2.1. Synthesis

The chemical structures of symmetric (1,6-**A**, 1,7-**A**, 1,6-**B**, and 1,7-**B**) and asymmetric PBIs (1-**A**, 1-**B**, 1,6-**C**, and 1,7-**C**) and their synthetic routes are shown in Scheme 1. The synthesis starts from an imidization of perylene bisanhydride (**PBA**) by reaction with cyclohexylamine. The cyclohexyl end-capping groups increase the steric bulkiness to the periphery of molecule, so that the solubility can be improved. The mononitration can then be achieved by a reaction of perylene bisimide (**PBI**) with cerium (IV) ammonium nitrate (CAN) and HNO_3_ under ambient temperature for 2 h, giving 1-**A** in high yields of *ca*. 95%. The presence of a single nitro substituent can be verified by the presence of seven signals at *δ* 8.1~8.8 ppm in the ^1^H-NMR spectrum and the strong absorption at 1539 cm^−1 in^ the FT-IR spectrum, corresponding to an asymmetrical stretching of the nitro group (Figure S1). Further nitration of 1-**A** using the same reagents at ambient temperature for 46 h gave dinitroperylene bisimides in 80% yield. Among the products, a 3:1 mixture of regioisomers (nitrated at the 1,6- or 1,7-positions) was observed by ^1^H-NMR spectroscopy, a situation similar to the result of bromination described previously by Rybtchinski, *et al*. [45]. The regioisomeric 1,6- and 1,7-dinitroperylene bisimides (1,6-**A** and 1,7-**A**) can be separated by high performance liquid chromatography (HPLC). Pure 1,7-regioisomer (1,7-**A**) can also be obtained through repetitive crystallizations.

**Figure 1:**
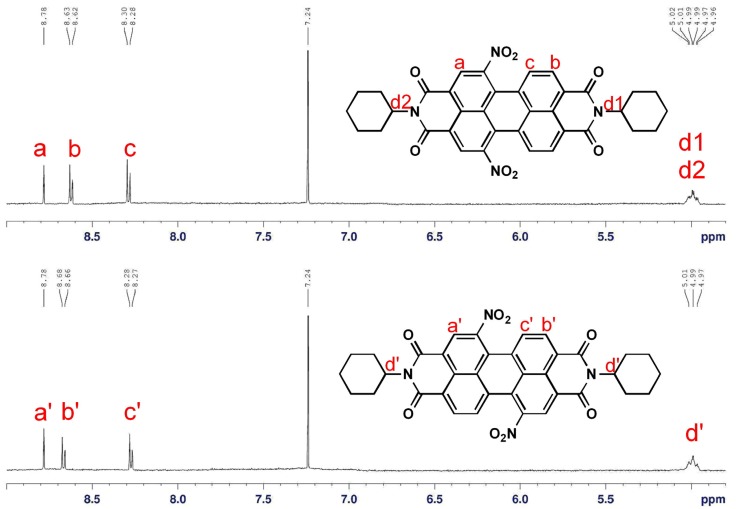
^1^H-NMR (500 MHz, CDCl_3_) partial spectra of regioisomerically pure perylene bisimides 1,6-**A** (top) and 1,7-**A** (bottom).

It is noteworthy that the characteristic signals of the regioisomers 1,6-**A** and 1,7-**A** in the ^1^H-NMR spectra (Figure 1), a singlet and two doublets of the perylene core protons, exhibit very small chemical shift value differences (0.01 and 0.04 ppm for the doublets at 8.26–8.68). However, a convenient unequivocal assignment of the NMR spectrum to the individual regioisomers 1,6-**A** and 1,7-**A** was performed on the basis of the signal of the cyclohexyl methine protons next to the imide nitrogen at 5.01 ppm. Because they are in the same chemical environment, both cyclohexyl methine protons of major regioisomer 1,7-**A** appear as one common multiplet at 5.01 ppm, but the signal splits into double multiplets for minor regioisomer 1,6-**A** (protons d1 and d2). Therefore, an unambiguous characterization has been made successfully on the basis of 500 MHz ^1^H-NMR.

**Figure 2:**
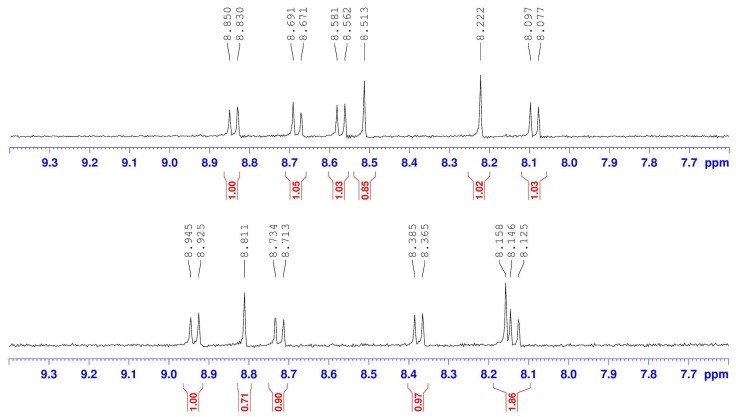
The ^1^H-NMR (400 MHz, CDCl_3_) spectra of 1,6-**C** (top) and 1,7-**C** (bottom).

The reduction of 1-nitro (1-**A**), 1,6-dinitro (1,6-**A**) and 1,7-dinitroperylene bisimides (1,7-**A**) by tin (II) chloride dihydrate (SnCl_2_·2H_2_O) in refluxing THF (6 h) afforded the 1-amino (1-**B**), 1,6-diamino (1,6-**B**) and 1,7-diaminoperylene bisimides (1,7-**B**), respectively. The symmetric structures of 1,6- and 1,7-diaminoperylene bisimides can be verified by the presence of three signals (one singlet and two doublet signals) at δ 8.0–9.0 ppm in the ^1^H-NMR spectrum, indicating that there are only three different kinds of protons in the perylene core. However, there are seven sets of signals, including six doublets and one singlet in the asymmetric 1-aminoperylene bisimide (Figure S2). On the other hand, the monoreduction of 1,6-**A** and 1,7-**A** can be performed by shortening the reaction time (1 h), giving asymmetric 1-amino-6-nitro (1,6-**C**) and 1-amino-7-nitroperylene bisimides (1,7-**C**), respectively. The asymmetric structures of 1-amino-6-nitro (1,6-**C**) and 1-amino-7-nitroperylene bisimides (1,7-**C**) can be verified by the presence of six signals (two singlet and four doublet signals) at δ 8.0–9.0 ppm in the ^1^H-NMR spectrum, which indicates that there are six different kinds of protons in the perylene core (Figure 2). Additionally, in the aromatic region four doublets and two singlets of the regioisomers 1,6-**C** and 1,7-**C** exhibit significant differences in chemical shift values (ca. 0.3 ppm). More importantly, these aromatic signals for 1,6-**C** appear in different order (doublet, doublet, doublet, singlet, singlet, doublet) compared to that of 1,7-**C** (doublet, singlet, doublet, doublet, singlet, doublet). This different pattern of appearance of singlets and doublets makes them easily recognizable by 400 MHz ^1^H-NMR.

### 2.2. Optical Properties

**Figure 3:**
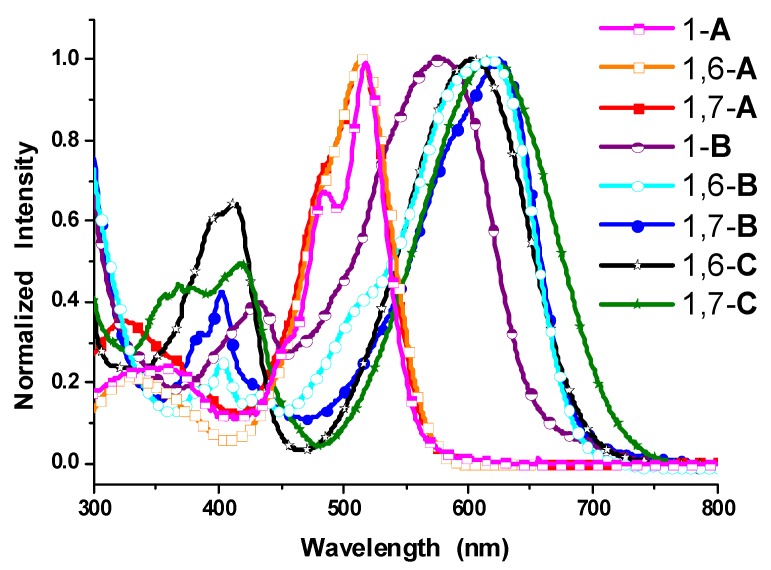
Normalized absorption spectra of 1-**A**, 1,6-**A**, 1,7-**A**, 1-**B**, 1,6-**B**, 1,7-**B**, 1,6-**C**, and 1,7-**C** in dichloromethane [53].

Figure 3 shows the steady state absorption spectra of **A**–**C** in dichloromethane. The longest wavelength absorption bands of 1-**A** and 1,6-**A** (1,7-**A**) appear at 518 nm and 515 nm, respectively. These peaks are assigned to the π-π* transitions localized on the perylene core (Figure S3). Moreover, the absorption spectra of all nitro-substituted PBIs (1-**A**, 1,6-**A**, and 1,7-**A**) are nearly identical with the spectrum of the non-substituted perylene bisimide (**PBI**), but they do not exhibit fluorescence. The reduction of 1,6-**A**/1,7-**A** (1-**A**) to 1,6-**B**/1,7-**B** (1-**B**) switches the substituent from an electron-withdrawing group to an electron-donating group and causes a large red shift. The spectra of monoamino-substituted (1-**B**) and diamino-substituted PBIs (1,6-**B** and 1,7-**B**) are dominated by very broad absorption bands that cover a large part of the visible spectrum (350–750 nm). These broad bands are representative for perylene bisimide derivatives *N*-substituted at the bay-core positions, due to a charge transfer absorption [49]. In contrast to 1,6-**A** and 1,7-**A**, the diamino-substituted PBIs 1,6-**B** and 1,7-**B** show significant differences in their absorptive features. In the case of 1,7-**B**, the lowest energy band is centered at 620 nm. Whereas for regioisomer 1,6-**B**, this longest wavelength absorption band is slightly broader and blue-shifted by ca. 6 nm with respect to that of 1,7-**B** and has a small shoulder at ca. 520 nm. The longest wavelength absorption band of both 1,6-**B** and 1,7-**B** is red-shifted in relation to that of the mono-substituted compound (1-**B**: 578 nm), which can be explained by the fact that the addition of amino (electron-donating) groups to the perylene core increases the HOMO energy level and hence decreases the energy gap. This viewpoint can be further supported by a theoretical approach based on density functional theory (see 2.3). On the other hand, the longest wavelength absorption band of the monoreduction adducts 1,6-**C** and 1,7-**C** appears at 603 nm and 616 nm, respectively, which is slightly blue-shifted relative to that of the corresponding direduction compounds 1,6-**B** (614 nm) and 1,7-**B** (620 nm). Careful examination of the absorption spectra of 1,6-**C** and 1,7-**C** also reveals that the S_0_ → S_1_ electronic transition band (absorption of up to 750 nm) and the S_0_ → S_2_ electronic transition band (absorption of up to 475 nm) of 1,7-**C** are both broader and red-shifted than those of 1,6-**C**. These features account for the differences in color observed by the naked eye.

### 2.3. Quantum Chemistry Computation

**Figure 4:**
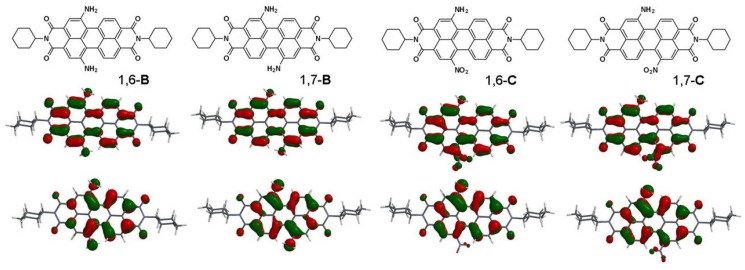
Computed frontier orbitals of 1,6-**B**, 1,7-**B**, 1,6-**C**, and 1,7-**C**. The upper graphs are the LUMOs and the lower ones are the HOMOs [53].

**Figure 5:**
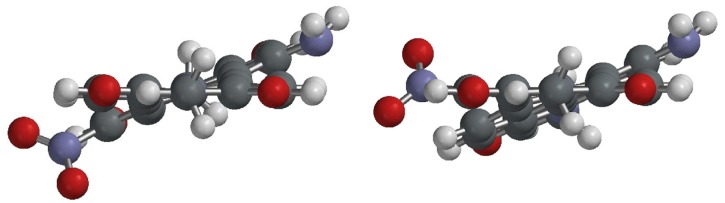
DFT (B3LYP/6-31G**) geometry-optimized structures of 1,6-**C** (left) and 1,7-**C** (right) shown with view along the long axis. For computational purposes, methyl groups replace the cyclohexyl groups at the imide positions.

To gain deeper insight into the molecular structures and electronic properties of **A**–**C**, quantum chemical calculations were performed using density functional theory (DFT) at the B3LYP/6-31G** level. The highest occupied molecular orbitals (HOMOs) and the lowest unoccupied molecular orbitals (LUMOs) of 1,6-**B**, 1,7-**B**, 1,6-**C**, and 1,7-**C** are shown in Figure 4. The HOMO of all amino-substituted PBIs (1,6-**B**, 1,7-**B**, 1,6-**C**, and 1,7-**C**) is delocalized mainly on the amino group and the perylene core. The LUMO of 1,6-**B**, and 1,7-**B** (1,6-diamino and 1,7-diamino) is delocalized from the central perylene core to the bisimide groups, while the LUMO of 1,6-**C** and 1,7-**C** (1-amino-6-nitro and 1-amino-7-nitro) is extended from the central perylene core to the peripheral nitro and the bisimide groups. The calculated and experimental parameters for perylene bisimide derivatives **A**–**C** are summarized in Table 1. The HOMO/LUMO energy levels of 1,6-**B** (1,7-**B**) are −5.43/−3.03 (−5.33/−3.05) eV, and those of 1-**B** are −5.62/−3.21 eV, yet both are higher than those of 1,6-**C** (−5.89/−3.55 eV) and 1,7-**C** (−5.92/−3.57 eV). This can be explained by the fact that the nitro (amion) substituent is an electron-withdrawing (−donating) group and hence decreases (increases) both the HOMO and LUMO energy levels. The relative band gap energies estimated from the longest absorption maxima of **A**–**C** are in good agreement with the theoretical calculations. The absorption spectra of 1-**B**, 1,6-**B**, 1,7-**B**, 1,6-**C**, and 1,7-**C** were also calculated by time-dependent DFT (TD-DFT) calculations (Franck–Condon principle, Table S1). The calculated excitation wavelengths for the S_0_ → S_1_ transitions are 547 nm for 1-**B**, 558 nm for 1,6-**B**, 579 nm for 1,7-**B**, 571 nm for 1,6-**C**, and 573 nm for 1,7-**C**, which are very close to the experimental results. The slight diversion between the experimental and calculated values results from the solvation effects for experiments and gas-phase for theoretical calculations. Furthermore, DFT (B3LYP/6-31G**) calculations show that the ground-state geometries of the perylene core have two core twist angles, *i*.*e*., approximate dihedral angles between the two naphthalene subunits attached to the central benzene ring; these are ~7.9° and ~15.9° for 1-**A**, ~17.2 and ~17.3° for 1,6-**A**, ~17.0° and ~17.1° for 1,7-**A**, ~9.2° and ~17.5° for 1-**B**, ~20.0 and ~20.1° for 1,6-**B**, ~19.2° and ~19.4° for 1,7-**B**, ~18.0 and ~19.3° for 1,6-**C**, and ~17.9° and ~18.5° for 1,7-**C** (Table 1 and Figure 5). The core twist angles of the 1,6-disubstituted (amino-substituted) PBIs are generally larger than those of 1,7-disubstituted (nitro-substituted) compounds.

**Table 1:** Calculated and experimental parameters for perylene bisimide derivatives.

Compound	HOMO ^a^	LUMO ^a^	*E*_g_^a^	*E*_g_^b^	Twisting angle (°)
1**-A**	−6.25	−3.84	2.41	2.39	7.9, 15.9
1,6**-A**	−6.55	−4.07	2.48	2.40	17.2, 17.3
1,7**-A**	−6.57	−4.11	2.46	2.40	17.0, 17.1
1**-B**	−5.62	−3.21	2.41	2.24	9.2, 17.5
1,6**-B**	−5.43	−3.03	2.40	2.13	20.0, 20.1
1,7**-B**	−5.33	−3.05	2.28	2.14	19.2, 19.4
1,6**-C**	−5.89	−3.55	2.34	2.19	18.0, 19.3
1,7**-C**	−5.92	−3.57	2.35	2.17	17.9, 18.5
**PBI**	−5.94	−3.46	2.48	2.38	0.0

^a^ Calculated by DFT/B3LYP (in eV); ^b^ At absorption maxima (*E*_g_ = 1240/λ_max_, in eV).

## 3. Experimental

### 3.1. General

The starting materials such as perylene-3,4,9,10-tetracarboxyldianhydride, cyclohexylamine, acetic acid, cerium (IV) ammonium nitrate (CAN), 1-methyl-2-pyrrolidinone (NMP), tetrahydrofuran (THF) and tin (II) chloride dihydrate (SnCl_2_·2H_2_O) were purchased from Merck (Whitehouse Station, NJ, USA), ACROS (Pittsburgh, PA, USA) and Sigma-Aldrich (St. Louis, MO, USA). Solvents were distilled freshly according to standard procedure. Column chromatography was performed using silica gel Merck Kieselgel *si* 60 (40–63 mesh). ^1^H-NMR spectra were recorded in CDCl_3_ on a Bruker 400 or 500 MHz instrument (Palo Alto, CA, USA). Mass spectra were recorded on a VG70-250S mass spectrometer (Tokyo, Japan). The absorption spectra were measured using a JASCO V-570 UV-Vis spectrophotometer (Tokyo, Japan). The Gaussian 03 program was used to perform the *ab initio* calculation on the molecular structure. Geometry optimizations for compounds **A**–**C** were carried out with the 6-31G** basis set to the B3LYP functional. Vibrational frequencies were also performed to check whether the optimized geometrical structures for all compounds were at energy minima, transition states, or higher order saddle points. After obtaining the converged geometries, the TD-B3LYP/6-31G** was used to calculate the vertical excitation energies.

### 3.2. Synthesis

#### 3.2.1. Synthesis of 1-Nitroperylene Bisimide (1-**A**)

Compound **PBI** (1.8 mmol), cerium (IV) ammonium nitrate (CAN, 1.2 g, 2.2 mmol), nitric acid (2.0 g, 31.7 mmol) and dichloromethane (150 mL) were stirred at 25 °C under N_2_ for 2 h. The mixture was neutralized with 10% KOH and extracted with CH_2_Cl_2_. After solvent was removed, the crude product was purified by silica gel column chromatography with eluent CH_2_Cl_2_ to afford 1-**A** in 95% yield. Characterization data: IR *v*_max_ 2928, 2851, 1700, 1659, 1596, 1539, 1401, 1336, 1262, 1245, 1190, 809, 743 cm^−1^; ^1^H-NMR (500 MHz, CDCl_3_) δ 8.74 (1H, d, *J* = 7.6 Hz,), 8.62~8.69 (4H, m), 8.55 (1H, d, *J* = 8.5 Hz), 8.18 (1H, d, *J* = 7.6 Hz), 5.00 (2H, m), 2.54 (4H, m), 1.91 (4H, m), 1.76 (6H, m), 1.47 (4H, m), 1.34 (2H, m); ^13^C-NMR (125 MHz, CDCl_3_) δ 163.4, 163.1, 163.0, 162.1, 147.6, 135.3, 132.7, 132.6, 131.2, 131.0, 129.3, 129.2, 128.9, 127.8, 127.4, 126.5, 126.4, 126.3, 126.2, 125.3, 124.6, 124.3, 123.9, 123.5, 54.5, 54.2, 29.1, 29.0, 26.5, 26.4, 25.3, 25.2; MS (FAB): *m/z* (relative intensity) 600 [M+H^+^, 100]; HRMS calcd. for C36H30O6N3 600.2135, found 600.2141.

#### 3.2.2. Synthesis of 1,6- and 1,7-Dinitroperylene Bisimides (1,6-A and 1,7-**A**)

Compound **PBI** (1.8 mmol), CAN (4.8 g, 8.8 mmol), nitric acid (8.0 g, 131.1 mmol) and dichloromethane (250 mL) were stirred at 25 °C under N_2_ for 48 h. The mixture was neutralized with 10% KOH and extracted with CH_2_Cl_2_. After solvent was removed, the crude product was purified by silica gel column chromatography with eluent CH_2_Cl_2_ to afford a mixture of 1,7- and -1,6-dinitroperylene bisimides, and ^1^H-NMR (400 MHz) analysis revealed a 3:1 ratio. Separation of the 1,6 and 1,7 isomers was performed on a preparative HPLC system equipped with a refractive index detector and fitted with a macro-HPLC column (Si, 8 μm, 250 × 22 mm). The eluent was 8:1 hexane/ethyl acetate flowing at 12 mL/min. Two fractions were collected from the column; the first was pure 1,6 isomer, and the second was pure 1,7 isomer. Characterization data: 1,6-**A**: ^1^H-NMR (500 MHz, CDCl_3_) δ 8.78 (2H, s), 8.63 (2H, d, *J =* 8.0 Hz), 8.30 (2H, d, *J =* 8.0 Hz), 5.01 (2H, m), 2.52 (4H, m), 1.90 (4H, m), 1.74 (6H, m), 1.46 (4H, m), 1.36 (2H, m); MS (FAB): *m/z* (relative intensity) 645 [M+H^+^, 100]; HRMS calcd. for C_36_H_29_O_8_N_4_ 645.1985, found 645.1983. Selected data for 1,7-**A**: ^1^H-NMR (500 MHz, CDCl_3_) δ 8.78 (2H, s), 8.68 (2H, d, *J =* 8.5 Hz), 8.28 (2H, d, *J =* 8.5 Hz), 5.01 (2H, m), 2.51 (4H, m), 1.92 (4H, m), 1.74 (6H, m), 1.46 (4H, m), 1.36 (2H, m); MS (FAB): *m/z* (relative intensity) 645 [M+H^+^, 100]; HRMS calcd. for C_36_H_29_O_8_N_4_ 645.1985, found 645.1981.

#### 3.2.3. Synthesis of 1-Aminoperylene Bisimide (1-**B**)

Tin chloride dihydrate (5.0 g, 22 mmol), and 1-**A** (1.0 g, 1.7 mmol) were suspended in THF (50 mL) and stirred 20 min. The solvent was refluxed 80 °C with stirring for 2 h. THF is removed at the rotary evaporator, and the residue was dissolved in ethyl acetate and washed with 10% sodium hydrate solution and brine. The organic layer was dried over anhydrous MgSO_4_ and the filtrate was concentrated under reduced pressure. The crude product was purified by silica gel column chromatography with eluent ethyl acetate/*n*-hexane (2/3) to afford 1-**B** in 80% yield. Characterization data: 1-**B**: IR *v*_max_ 3346, 3240, 2926, 1694, 1653, 1372, 1338, 1260, 806, 747 cm^−1^; ^1^H-NMR (400 MHz, CDCl_3_) δ 8.62 (1H, d, *J* = 8.0 Hz), 8.45 (1H, d, *J* = 7.6 Hz), 8.38 (1H, d, *J* = 8.0 Hz), 8.25 (1H, d, *J* = 7.6 Hz), 8.18 (1H, d, *J* = 8.0 Hz), 8.10 (1H, d, *J* = 8.0 Hz), 7.98 (1H, s), 5.03, (2H, s), 4.99 (2H, m), 2.55 (4H, m), 1.91 (4H, m), 1.74 (6H, m), 1.46~1.40 (6H, m); MS (FAB): *m/z* (relative intensity) 570 [M+H^+^, 100]; HRMS calcd. for C_36_H_32_O_4_N_3_ 570.2393, found 570.2396.

#### 3.2.4. Synthesis of 1,6- and 1,7-Diaminoperylene Bisimides (1,6-**B** and 1,7-**B**)

Tin chloride dihydrate (1.0 g, 4.8 mmol), 1,6- or 1,7-dinitroperylene bisimides (0.5 g, 0.8 mmol) were suspended in THF (50 mL), and stirred at 25 °C under N_2_ for 20 min. The solvent was refluxed 80 °C with stirring for 6 h. THF is removed at the rotary evaporator, and the residue was dissolved in ethyl acetate and washed with 10% sodium hydrate solution and brine. The organic layer was dried over anhydrous MgSO_4_ and the filtrate was concentrated under reduced pressure. The crude product was purified by silica gel column chromatography with eluent ethyl acetate/*n*-hexane (4/5) to afford 1,6- or 1,7-diaminoperylene bisimides 1,6-**B** or 1,7-**B** in 82% yield. Characterization data: 1,6-**B**: ^1^H-NMR (400 MHz, CDCl_3_) δ 8.77 (2H, d, *J* = 8.0 Hz), 8.51 (2H, d, *J* = 8.0 Hz), 7.85 (2H, s), 5.05 (2H, m), 4.98 (4H, s), 2.59 (4H, m), 1.92 (4H, m), 1.76 (6H, m), 1.27–1.56 (6H, m); MS (FAB): *m/z* (relative intensity) 585 [M+H^+^, 100]; HRMS calcd. for C_36_H_3__3_O_4_N_4_ 585.2502, found 585.2508. Selected data for 1,7-**B**: ^1^H-NMR (400 MHz, CDCl_3_) δ 8.90 (2H, d, *J* = 8.0 Hz), 8.25 (2H, d, *J* = 8.0 Hz), 8.14 (2H, s), 5.04, (2H, m), 4.94 (4H, s), 2.61 (4H, m), 1.93 (4H, m), 1.74 (6H, m), 1.36–1.54 (6H, m); MS (FAB): *m/z* (relative intensity) 585 (M+H^+^, 100); HRMS calcd. for C_36_H_3__3_O_4_N_4_ 585.2502, found 585.2504.

#### 3.2.5. Synthesis of 1-Amino-6-nitro and 1-Amino-7-nitroperylene Bisimides (1,6-**C** and 1,7-**C**)

Tin chloride dihydrate (0.6 g, 3.6 mmol), 1,6- or 1,7-dinitroperylene bisimides (0.4 g, 0.6 mmol) were suspended in THF (50 mL), and stirred at 25 °C under N_2_ for 20 min. The solvent was refluxed 80 °C with stirring for 1 h. THF is removed at the rotary evaporator, and the residue was dissolved in ethyl acetate and washed with 10% sodium hydrate solution and brine. The organic layer was dried over anhydrous MgSO_4_ and the filtrate was concentrated under reduced pressure. The crude product was purified by silica gel column chromatography with eluent ethyl acetate/*n*-hexane (2/3) to afford 1-amino-6-nitro or 1-amino-7-nitroperylene bisimides (1,6-**C** or 1,7-**C**) in 80%. Characterization data: 1,6-**C**: ^1^H-NMR (400 MHz, CDCl_3_) δ 8.85 (1H, d, *J* = 8.0 Hz), 8.69 (1H, d, *J* = 8.0 Hz), 8.58 (1H, d, *J* = 8.0 Hz), 8.51 (1H, s), 8.22 (1H, s), 8.09 (1H, d, *J* = 8.0 Hz), 5.31, (2H, s), 5.02 (2H, m), 2.54 (4H, m), 1.93 (4H, m), 1.76 (6H, m), 1.40–1.51 (6H, m); MS (FAB): *m/z* (relative intensity) 615 [M+H^+^, 100]; HRMS calcd. for C_3__6_H_31_O_6_N_4_ 615.2244, found 615.2246. Characterization data: 1,7-**C**: ^1^H-NMR (400 MHz, CDCl_3_) δ 8.95 (1H, d, *J* = 8.0 Hz), 8.81 (1H, s), 8.73 (1H, d, *J* = 8.4 Hz), 8.39 (1H, d, *J* = 8.0 Hz), 8.16 (1H, s), 8.15 (1H, d, *J* = 8.4 Hz), 5.33, (2H, s), 5.07 (2H, m), 2.55 (4H, m), 1.94 (4H, m), 1.77 (6H, m), 1.36–1.54 (6H, m); MS (FAB): *m/z* (relative intensity) 615 [M+H^+^, 100]; HRMS calcd. for C_3__6_H_31_O_6_N_4_ 615.2244, found 615.2240.

## 4. Conclusions

We have successfully synthesized, separated, and characterized 1,6- and 1,7-regioisomers of asymmetric (1,6-**C/**1,7-**C**) and symmetric PBIs (1,6-**A/**1,7-**A** and 1,6-**B/**1,7-**B**). The regioisomers of dinitro-substituted PBIs (1,6-**A** and 1,7-**A**) were separated by conventional high performance liquid chromatography. Subsequently, the reduction of 1,6-**A** and 1,7-**A** afforded the corresponding diaminoperylene bisimides 1,6-**B** and 1,7-**B**, respectively. On the other hand, the monoreduction of 1,6-**A** and 1,7-**A** can be executed by reducing the reaction time, giving asymmetric 1-amino-6-nitro (1,6-**C**) and 1-amino-7-nitroperylene bisimides (1,7-**C**), respectively. To our best knowledge, this is the first time the asymmetric 1,6-disubstituted perylene bisimide 1,6-**C** has been obtained in pure form. Our studies have also shown that these 1,6- and 1,7-isomers can readily be characterized by 500 MHz ^1^H-NMR. The optical properties of 1,6-**A** and 1,7-**A** were found to be virtually the same. However, the regioisomers 1,6-**C** and 1,7-**C**, as well as 1,6-**B** and 1,7-**B**, exhibit significant differences in their optical features; the S_0_ → S_1_ and the S_0_ → S_2_ electronic transition bands of 1,7-**C** are both broader and red-shifted than those of 1,6-**C**, while the absorption spectrum of 1,6-**B** covers a large part of the visible region relative to that of 1,7-**B**. The results offer the potential to synthesize 1,6-disubstituted perylene bisimide derivatives with extended optical properties. Working toward their applications on *n*-type organic semiconductors (1,6-**A** and 1,7-**A**) and organic photovoltaics (1,6-**B**/1,7-**B** and 1,6-**C**/1,7-**C**) is in progress.

## Supplementary Materials

Supplementary materials can be accessed at: http://www.mdpi.com/1420-3049/19/1/327/s1.
