# Supplementary material for: 1,6- and 1,7-Regioisomers of Asymmetric and Symmetric Perylene Bisimides: Synthesis, Characterization and Optical Properties
Source: Molecules. 2013 Dec 27;19(1):327–41. doi: 10.3390/molecules19010327 (PMC6270995; doi:10.3390/molecules19010327)
Supplement: Supplementary file 1 [file molecules-19-00327-s001.pdf]

Supplementary Materials

Figure S1. FT-IR spectra of **PBI** (dashed line) and 1-**A** (solid line).

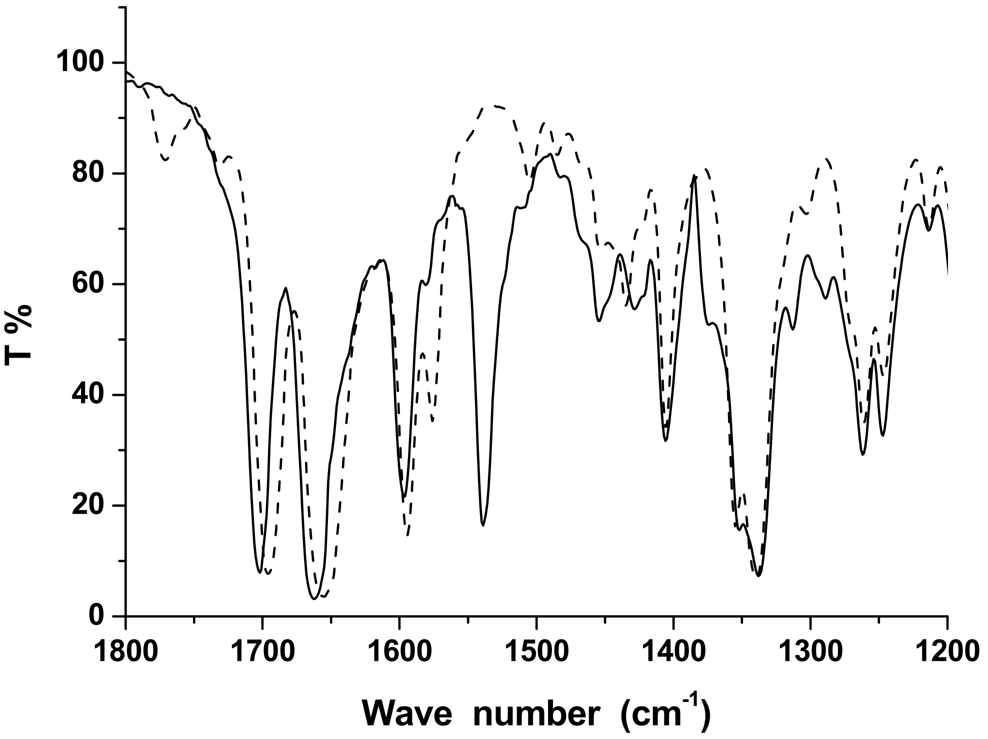

Figure S2. The ^{1}H-NMR (400 MHz, $CDCl_3$) spectra of 1-**B**.

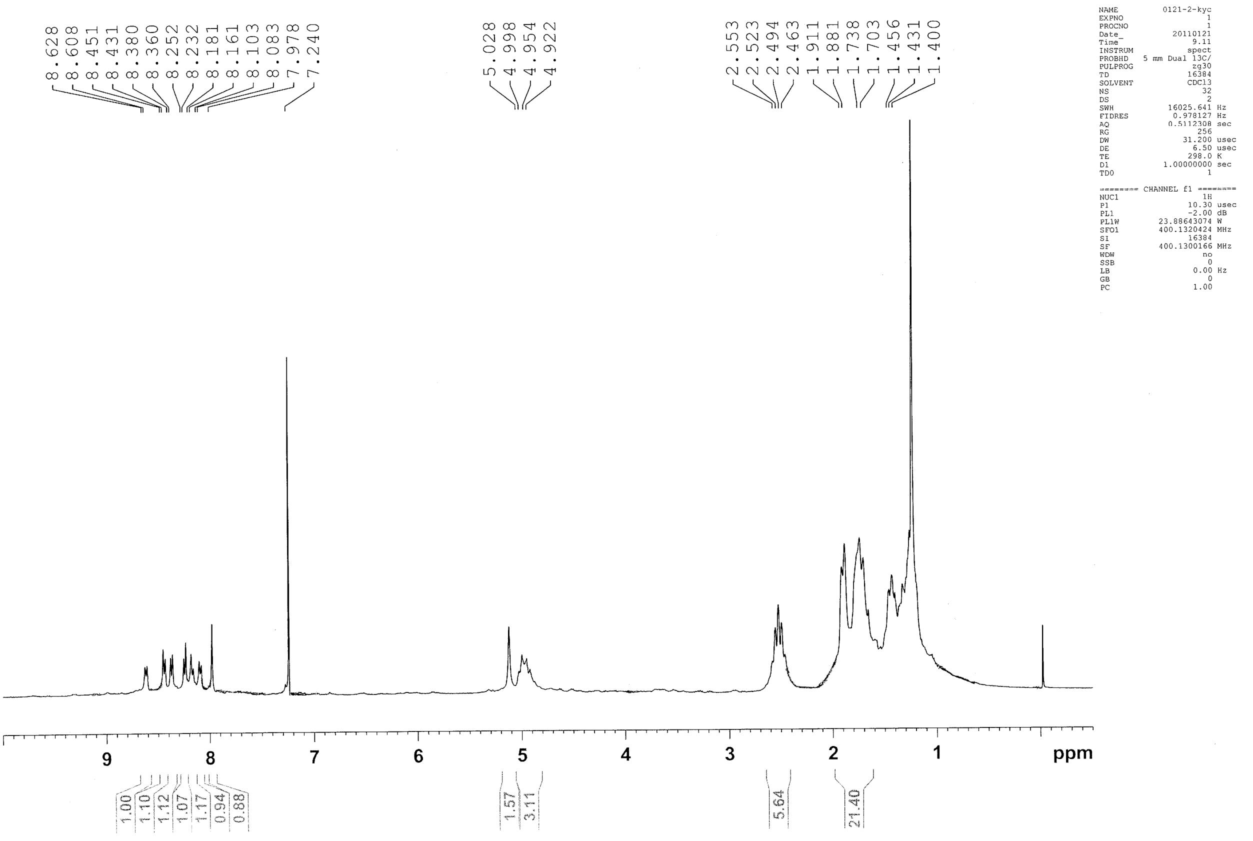

Figure S3. Computed frontier orbitals of 1,6-**A** and 1,7-**A**. The upper graphs are the LUMOs and the lower ones are the HOMOs.

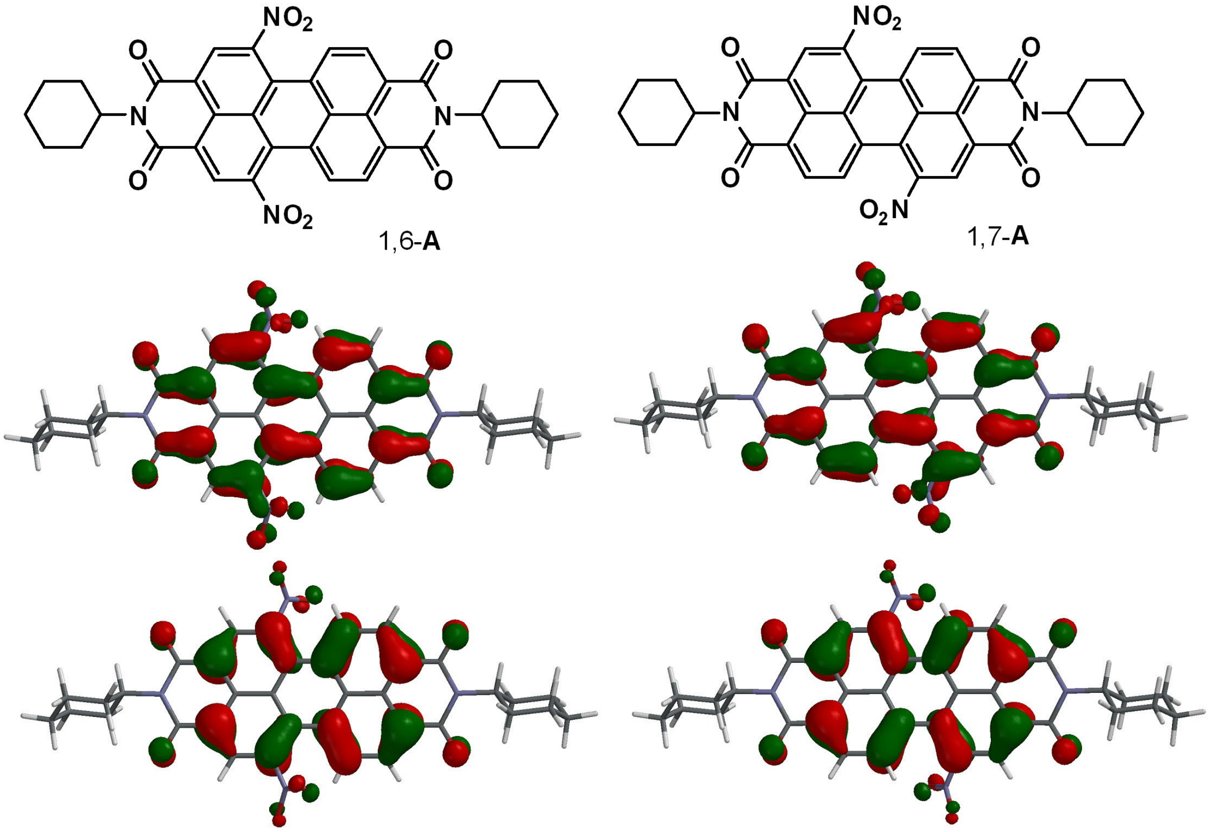

Figure S4. DFT (B3LYP/6-31G**) geometry-optimized structures of 1,6-**B** (left) and 1,7-**B** (right) shown with view along the long axis. For computational purposes, methyl groups replace the cyclohexyl groups at the imide positions.

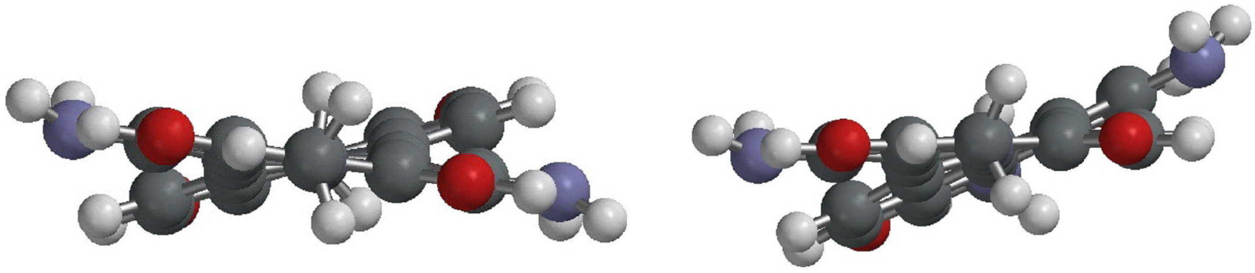

Table S1. Selected electronic excitation energies and corresponding oscillator strengths (*f*), main configurations, and CI coefficients of the low-lying electronically excited states of **B** and **C** [a].

Compound	Singlet	Electronic transition	Energy	*f*	Composition [b]	CI [c]
1,6-C	UV–Vis	$S_0 \rightarrow S_1$<br>$S_0 \rightarrow S_2$	2.17 eV/571 nm<br>2.97 eV/417 nm	0.4161<br>0.1133	H→L<br>H-1→L<br>H→L+1	0.70363<br>0.18917<br>0.66735
1,7-C	UV–Vis	$S_0 \rightarrow S_1$<br>$S_0 \rightarrow S_2$	2.16 eV/573 nm<br>2.97 eV/418 nm	0.4947<br>0.0304	H→L<br>H-1→L<br>H→L+1	0.70363<br>0.64469<br>0.24965
1,6-B	UV–Vis	$S_0 \rightarrow S_1$<br>$S_0 \rightarrow S_2$	2.22 eV/558 nm<br>2.74 eV/452 nm	0.5806<br>0.0692	H→L<br>H-1→L<br>H→L+4	0.70663<br>0.69227<br>0.11954
1,7-B	UV–Vis	$S_0 \rightarrow S_1$<br>$S_0 \rightarrow S_2$	2.14 eV/579 nm<br>2.95 eV/420 nm	0.5645<br>0.0036	H→L<br>H-1→L<br>H→L+1	0.70753<br>0.68128<br>0.12636
1-B	UV–Vis	$S_0 \rightarrow S_1$<br>$S_0 \rightarrow S_2$	2.26 eV/547 nm<br>3.11 eV/399 nm	0.6165<br>0.1002	H→L<br>H-1→L	0.70581<br>0.68061

[a] Calculated by TDDFT/B3LYP/6-31G**; [b] H stands for HOMO and L stands for LUMO. Only the main configurations are presented; [c] CI coefficients are in absolute values.
